# Ceftolozane/tazobactam for the treatment of bacteremia: a systematic literature review (SLR)

**DOI:** 10.1186/s12941-022-00528-0

**Published:** 2022-10-03

**Authors:** Z. S. Khankhel, R. J. Dillon, M. Thosar, C. Bruno, L. Puzniak

**Affiliations:** 1 PrecisionHEOR, Boston, MA USA; 2 Merck & Co., Inc, Kenilworth, NJ USA

**Keywords:** Bacteremia, Blood stream infection, Clinical cure, Microbiological cure, Mortality

## Abstract

**Background:**

Bloodstream infections (BSIs), or bacteremia, are responsible for considerable disease burden. Increasing rates of antibiotic resistance and delays in selection of appropriate treatment lead to increased morbidity, mortality, and costs. Due to limitations of current standard treatments, especially for bacteremia caused by resistant pathogens, a systematic literature review (SLR) was conducted to understand the utilization of ceftolozane/tazobactam (C/T) in bacteremia.

**Methods:**

Electronic database searches of EMBASE^®^, MEDLINE^®^, CCTR and Northern Lights, as well as hand searches of conference proceedings from the last two annual meetings (i.e., 2018, 2019) of the European Congress of Clinical Microbiological and Infectious Diseases (ECCMID) and the Infectious Diseases Society of America’s annual meeting (IDWeek) were conducted. A total of 23 studies reporting on patients with bacteremia receiving C/T were included in the review.

**Results:**

Most studies were observational (k = 20 studies), though few interventional studies were also identified (k = 3). Heterogeneity was ubiquitous with respect to source of bacteremia (i.e., primary or secondary), source of infection (for secondary bacteremia), pathogen type, antibiotic resistance, C/T dose, and outcome definitions. This heterogeneity, along with limited data, and small sample sizes (n = 1 to 31) made it difficult to draw any substantial conclusions, though overall results were favorable to C/T with respect to the outcomes of interest. Nineteen studies reported clinical cure or success (primary bacteremia: k = 6, reported range: 33.3% to 100%; secondary bacteremia: k = 8, 60% to 100%; mixed/unspecified bacteremia: k = 10, 50% to 91.7%). Eight studies reported microbiological cure or eradication rates (primary: k = 3, all reporting 100%; secondary: k = 4, 68% to 80%; mixed/unspecified: k = 5, 60% to 80%). Thirteen studies reported mortality (primary: k = 4, 0% to 14%; secondary: k = 7, 0% to 100%; or mixed/unspecified bacteremia: k = 7, 0% to 51.6%). One study each also reported composite clinical response, relapse, hospital re-admission, and hospital length of stay.

**Conclusions:**

Although the available evidence and observed trends for C/T in bacteremia should be interpreted with caution, the direction of effect would support the utilization of C/T for these difficult to treat infections. Future research should supplement the existing evidence by considering the impact of key treatment effect modifiers without contributing to the observed heterogeneity.

**Supplementary Information:**

The online version contains supplementary material available at 10.1186/s12941-022-00528-0.

## Background

Bloodstream infections (BSIs), also referred to as bacteremia, are responsible for considerable disease burden worldwide with incidence estimated to be 189 per 100,000 persons in the United States (US) and to range between 168 and 220 persons per 100,000 across various Northern European nations [1–3]. BSIs or positive blood cultures can be considered primary (i.e., the sole source of infection) or secondary to another source of infection, such as the respiratory tract. Both gram-positive (e.g. *Enterococcus, Staphylococcus*, *Streptococcus*) and gram-negative pathogens (e.g.*, Escherichia coli, Klebsiella, Pseudomonas)* may cause bacteremia; the infection tends to be more prevalent among populations that have weaker immune systems, are critically ill, and/ or have multiple comorbidities [4, 5].

Increasing rates of antimicrobial resistance, especially to fluroquinolones, cephalosporins and carbapenems, pose significant challenges to the management of bacteremia. Further, many of the pathogens causing bacteremia are multi-drug resistant (MDR), extensively-drug resistant (XDR), or pan drug-resistant (PDR). Delays in appropriate treatment of bacteremia are associated with increasing morbidity, mortality, and costs [1–3]. To optimize management of the infection it is imperative to promptly identify the causative pathogen, susceptibility status, and physical source(s) of infection. Blood cultures are key to facilitating selection of an appropriate treatment for specific pathogen and susceptibility profiles.

Ceftolozane/tazobactam (C/T) is a combination of a novel antipseudomonal cephalosporin and an established β-lactamase inhibitor. It is approved by the US Food and Drug Administration (FDA) and the European Medicines Agency (EMA) for the treatment of complicated urinary tract infections (cUTIs) including pyelonephritis, hospital-acquired bacterial pneumonia and ventilator-associated bacterial pneumonia (HABP/VABP), and in combination with metronidazole in complicated intra-abdominal infections (cIAIs) [6, 7]. Due to the lack of viable treatment options for bacteremia, especially caused by resistant pathogens, a systematic literature review (SLR) conducted in accordance with established guidance was undertaken to understand the impact of C/T in primary or secondary bacteremia.

## Methods

All publications were identified and evaluated for inclusion using predefined selection criteria based on the Population, Interventions, Comparisons, Outcomes, Time and Study design (PICOTS) structure [8, 9]. The full PICOTS criteria are presented in Additional File 1.

### Search strategy

Electronic database searches of EMBASE®, MEDLINE®, CCTR and Northern Lights via the OVID® platform were conducted. Searches included intervention terms only and were conducted from database inception to February 2020. In addition, manual searches of the European Congress of Clinical Microbiological and Infectious Diseases (ECCMID) annual meeting and the Infectious Diseases Society of America’s IDWeek were carried out for 2018 and 2019. Only articles published in English were evaluated. The full search strategy is available in an Additional File 2.

### Study selection

Study selection occurred in two stages based on review of the titles and abstracts (stage I) and then, full-text screening (stage II). Full-text articles satisfying eligibility criteria were included in the SLR and underwent data extraction. During both stages of study selection two, independent researchers reviewed each publication. Discrepancies between researchers were resolved by discussion, with the support of a third, more senior investigator, as needed.

### Data extraction

Data pertaining to study characteristics and methods, patient, and treatment characteristics, as well as the outcomes of interest were captured from included studies. Outcomes were extracted as reported based on data availability. All outcomes’ definitions were collected as reported by the authors of the included studies. If only two of the following were reported, the remaining data point was calculated: number evaluated, number experiencing outcome, percent of patients experiencing outcome. In instances where patients with mixed infections were evaluated, values for outcomes specific to bacteremia patients were derived based on data reported. Individual patient data available from case series or other study designs that reported data for single patients but not an entire population were pooled together to represent a group of bacteremia patients, data permitting.

As with study selection, data were extracted by a single researcher and independently validated by a second researcher; these were the same persons involved in study selection. Any discrepancies were resolved by discussion between reviewers, including a third, if needed. Prior to reporting, the full dataset was quality checked by two independent reviewers. Data were stored and managed in a Microsoft Excel^®^ workbook.

## Results

### Overview of evidence base

After removing duplicate citations, a total of 1,010 unique titles/abstracts were identified from the database searches, of which 94 progressed to full-text review. After full-text review, 21 were included in the SLR. Three publications were also identified from the grey literature searches of conference proceedings. Ultimately, 24 publications on 23 unique studies were included in the SLR. An overview of the literature selection process is presented in the PRISMA Diagram (see Additional File 3).

The 23 studies included in the SLR were of varying design and were conducted in various geographic settings. Cohort studies were the most commonly utilized study type (k = 13 retrospective cohort studies, [10–22] one prospective cohort study [23]), followed by case reports/series (k = 2 case reports [24, 25], three case series [26–28], one case–control study [29]), and clinical trials (k = 2 randomized controlled trials [RCTs], [30, 31] one single-arm trial [32]). Most studies were conducted in the US (k = 10) [10, 11, 17–20, 26–28, 33] and Spain (k = 6^15,18,21,25–27^); three were conducted internationally [21, 30, 31], two in Italy [12, 24], one in Japan [32], and one in Saudi Arabia [13]. The studies also varied with respect to clinical setting; with seven conducted in medical centers [15, 18, 20, 22, 26–28], thirteen conducted in hospitals [10–14, 16, 19, 21, 23–25, 29, 30], one conducted in multicenter health systems [17], and two conducted at unspecified clinical sites [31, 32]. Sample size of overall populations evaluated in the included studies ranged from one [13, 14, 19, 22, 24, 25] to 398 [31] patients. However, six studies reported on patients with mixed infections [11, 12, 14, 17, 18, 21]. Therefore, the sample size specific to patients with primary (n = 1 to 7 patients), secondary (n = 1 to 25 patients), and mixed or unspecified (n = 1 to 31 patients) bacteremia tended to be smaller and a subset of the overall population.

Heterogeneity was ubiquitous across the evidence base with respect to source of bacteremia (i.e., primary or secondary), source of infection (for secondary bacteremia), pathogen type, antibiotic resistance, C/T dose, and outcome definitions. The observed heterogeneity was still present after stratifying results by primary, secondary, or mixed/unspecified bacteremia. Outcome assessment time points also varied across the evidence base but were rarely reported. Variation in the evaluated populations and C/T dose are discussed further below. Detailed outcome definitions are presented in Additional File 4.

#### Pathogen type

The causative pathogen was described in the majority of studies (k = 20/23) [10–27, 29, 30, 33]. *Pseudomonas aeruginosa* was the most frequently reported pathogen (k = 13/23) [11–15, 18–21, 23, 26, 29]. Six studies evaluated patients with mixed/polymicrobial infections [10, 16, 24, 25, 27, 30], five [10, 16, 25, 27, 30] *Pseudomonas aeruginosa* in combination with other pathogens [10, 16, 25, 27, 30], and one joint *E. coli* and *K. pneumonia* infection [24].

#### Antibiotic resistance

Pathogen susceptibility profiles were also described in the majority of the studies (k = 16/23) [10–18, 22, 23, 25–29]. Nine evaluated patients with MDR infections (all 100% MDR) [10–18, 20, 22, 23, 25, 26, 28, 29, 33] and seven evaluated a mixed patient population with either MDR, XDR, and/or PDR infections (five mixed MDR/XDR [12, 16, 20, 23, 29]; two mixed MDR/PDR/XDR [22, 28]).

#### Underlying infection for secondary bacteremia

Of the 13 [11, 13, 14, 17–21, 25, 26, 30–32] studies reporting on a secondary bacteremia population there was variability in the underlying infection source(s). Six (k = 6/13) studies evaluated patients with mixed underlying infections including a combination of biliary, bone/joint, central-line, intra-abdominal, left-ventricular assist device, otitis and mastoiditis, perianal abscesses, pyelonephritis, respiratory, submandibular fasciitis, surgical site, urinary tract infection (UTI), and/or wound. Five of the remaining studies (k = 5/13) evaluated patients with a single underlying infection and two (k = 2/13) did not specify infection source. Of the five studies reporting a single infection source, one study each evaluated patients with lower perianal abscess [13], pneumonia [18] or nosocomial pneumonia [30], respiratory tract infections [21], and skin and soft tissue infections [25]. Among the two studies not reporting infection source, one evaluated hematopoietic‑cell transplant recipients or those with a hematologic malignancy [26] and another evaluated maternal sepsis patients [24].

#### C/T dose

Administered dose of C/T was reported in all the publications and ranged from 0.375 g [13, 18] to 3 g [26]; the most frequently administered doses were 1.5 g (k = 14) [10, 12–16, 18, 19, 21, 24, 25, 28, 31, 32] and 3.0 g IV 8qh (k = 9) [10, 11, 14–16, 18–20, 30]. Actual treatment duration ranged from a minimum of 3 days [14] to a maximum 48 days [18] and frequency of administration was reported to be every 8 h in all but two studies, which reported a single daily bolus of C/T [27, 31]. In addition to C/T, concomitant therapy with amikacin, colistin or metronidazole was administered in six [11, 13, 19, 22, 24, 29] of the 23 studies.

### Clinical cure or success

Nineteen studies reported clinical cure or success rates among patients with either primary bacteremia (k = six) [11, 12, 15, 17, 18, 23, 33], secondary bacteremia (k = 8) [11, 13, 14, 18, 19, 25, 26, 30] and/or mixed/unspecified bacteremia (k = 10) [10–12, 14, 16, 22, 24, 27–29]. Fifteen of the 19 studies defined clinical cure or success, three of which relied on a resolution of signs and symptoms present at diagnosis or baseline and nine of which expanded this definition with additional criteria, including fever reduction, improved imaging (details related to imaging not specified), in-hospital survival, lack of microbiological evidence of infection, lack of recurrence, no new signs or symptoms, and/or no use additional antibiotic therapy. For two studies [18, 23] clinical cure or success was determined as the inverse of clinical failure and a single study relied on repeat microbiological clearance to define clinical success or failure [10].

#### Primary bacteremia

Six retrospective cohort studies evaluated patients with primary bacteremia [11, 12, 15, 17, 18, 23] and reported clinical cure or success rates ranging from 33.3% [23] to 100% (n = 1 to 7 patients) (see Table 1) [12, 15, 17]. In the study reporting 33% clinical success, 86.2% of the infections were caused by an XDR pathogen, compared with 0% to 50.5% of infections caused by an XDR pathogen in the remaining five studies. Upon removing this study, the range of clinical success was 86% [11] to 100% (n = 3 to 7 patients) [12, 15, 17].

**Table 1:** Clinical cure or success in primary bacteremia patients receiving C/T

Author, year	Study design	Pathogen type	Antibiotic resistance	Outcome definition	Time point	% (n/N) Reporting clinical cure or success
Retrospective cohort studies						
Bassetti et al., 2019 [12]	Retrospective cohort	*Pseudomonas*: 100% (not further specified)	MDR infection: 17.8%, XDR: 50.5%, PDR infection: 2%^a^	Complete resolution of clinical signs and symptoms related to P. aeruginosa infection and lack of microbiological evidence of infection	–	100% (6/6)
Diaz-Canestro et al., 2018 [23]	Retrospective cohort	*Pseudomonas*: 100% (not further specified)	MDR: 10.3%, XDR: 86.2%^a^	Derived from clinical failure defined as persistent signs or symptoms of infection and positive culture after 7 days of treatment	–	33.3% (1/3)
Elabor et al., 2018 [15]	Retrospective cohort	*Pseudomonas*: 100% (not further specified)	MDR infection: 100%	Resolution of signs and symptoms present on diagnosis	–	100% (4/4)
Gallagher et al., 2018 [17]	Retrospective cohort	*Pseudomonas*:100% (not further specified)	MDR infection: 100%	Defined as improved signs and symptoms from baseline to the end of therapy with fever reduction	–	100% (6/6)
Haidar et al., 2017 [18]	Retrospective cohort (single patient)	*P. aeruginosa*:100%	MDR infection: 100%	Clinical failure was defined as attributable mortality due to P. aeruginosa, persistent signs or symptoms of infection or positive culture despite ≥ 7 days of C/T, or recurrent P. aeruginosa infection (recurrent signs and symptoms and recurrent culture positivity within 90 days)^a^	–	100% (1/1)^b^
King et al., 2018 [11]	Retrospective cohort	*Pseudomonas*: 100% (not further specified)	MDR infection: 100%	Improved symptoms, improved imaging where relevant, and fever reduction	–	86.0% (6/7)

*MDR* Multi drug resistant, *PDR* Pan drug resistant, *XDR* Extensively drug resistant

^a^The antibiotic resistance data captured here is not bacteremia specific

^b^An extracted number of 1 denotes clinical success

#### Secondary bacteremia

Five retrospective cohort studies [11, 13, 14, 18, 19], one case series [26], one case report [25], and one RCT [30] also reported on clinical cure or success in patients with secondary bacteremia (see Table 2). Clinical cure or success ranged from 0% [13] to 100% [18] (n = 1 to 18 patients) across the five retrospective studies; removing a study reporting on only one patient the value ranged from 60% [14] to 100% [18] (n = 2 to 18 patients). Four (k = 4/5) retrospective studies also reported clinical cure or success by infection source: one evaluated only patients with pneumonia[22], one evaluated patients with various infections and provided individual patient data (IPD) for each infection source [13, 14], and two evaluated patients with various infections and provided data for smaller subgroups of patients with specific infection source(s) [11, 18, 19] (see Additional File 5). In the study providing IPD, clinical cure or success was achieved by the one patient with abdominal infection (100%, 1/1) and one patient with venous central catheter infection (100%, 1/1); only one of three patients (33.3%, 1/3) with respiratory infection reported clinical cure [14] (see Additional File 5). Across the two studies providing data for subgroups of patients with specific infections, both found pneumonia patients to have lower rates of clinical cure or success than patients with other infection types. In the first study, clinical cure or success was lower among patients with wound infection (0%, 1/1) and pneumonia (38% [3/8]), compared with UTI (86%, 6/7) and intra-abdominal infections (100%, 1/1) [11] (see Additional File 5). In the second study, clinical cure or success was lower among patients with pneumonia (33%, 1/3), compared with 100% (1/1) for each: central-line associated BSI, left-ventricular assist device infection, and pyelonephritis [19] (see Additional File 5). The rate for clinical cure or success reported specifically for pneumonia patients across these retrospective studies (33% to 38%) was similar to the rate reported among patients with nosocomial pneumonia in an RCT comparing C/T with meropenem: 36% (of 25 enrolled patients) [30].

**Table 2:** Clinical cure or success in secondary bacteremia patients receiving C/T

Author, year	Study design	Source	Pathogen type	Antibiotic resistance	Outcome definition	Time point	% (n/N) Reporting clinical cure or success
Case report and case series							
Hakki and Lewis et al., 2018 [26]	Case series	NR^a^	*Pseudomonas:* 100% (not further specified)	MDR infection: 100%	Defined as resolution of signs and symptoms of the infection during treatment with C/T, clearance of bacteremia (if present) within 72 h of initiation of C/T, and absence of infection recurrence, defined as signs and symptoms of infection along with culture positivity for P. aeruginosa while receiving C/T or within 30 days of completion of C/T therapy	30 days	66.7% (2/3)
Sousa Dominguez et al., 2017 [25]	Case report	Skin and soft-tissue infection	*Pseudomonas:* 100% (not further specified), *Streptococcus:* 100%, *K. pneumonia:* 100%^b^	MDR infection: 100%	–	–	100% (1/1)
Randomized controlled trial (RCT)							
Kollef et al. et al., 2019 [30]	RCT	Nosocomial pneumonia	*Pseudomonas:* 17.4% (Not further specified), *Enterobacter:* 53.86%^b^	–	Defined as: 1) Complete resolution with no new signs of ventilator-associated nosocomial pneumonia [VNP), which were present at baseline 2) No new signs, symptoms or complications attributable to VNP 3) No additional antibiotic therapy administered for VNP, except for the approved adjunctive therapy 4) Patient is alive	–	36.0% (9/25)
Retrospective cohort studies							
Bosaeed et al., 2020 [13]	Retrospective cohort (single patient)	Complicated perianal abscesses	*Pseudomonas:* 100% (not further specified)	MDR infection: 100%	Clinical success was based on microbiological clearance (whenever repeated cultures were available; clinical resolution of signs and symptoms of infection; and 30-day in-hospital survival after initiation of C/T treatment)	14 days	0% (0/1)
Caston et al., 2017 [14]	Retrospective cohort	Mixed infections: venous central catheter (N = 1), respiratory (N = 3) and abdominal (N = 1)	*Pseudomonas:* 100% (not further specified)	MDR infection: 100%	Clinical outcome considered a "cure" when attending physician observed a resolution of signs and symptoms and there were no radiologic findings of infection	30 days (after isolation of P. aeruginosa)	60.0% (3/5)
Haidar et al., 2017 [18]	Retrospective cohort	Pneumonia	*P. aeruginosa:* 100%	MDR infection: 100%	Clinical failure was defined as attributable mortality due to P. aeruginosa, persistent signs or symptoms of infection or positive culture despite ≥ 7 days of C/T, or recurrent P. aeruginosa infection (recurrent signs and symptoms and recurrent culture positivity within 90 days)	–	100% (2/2)
King et al., 2018 [11]	Retrospective cohort	Mixed infections: Pneumonia (N = 8), UTI (N = 7), intra-abdominal (N = 4), wound (N = 1)	*Pseudomonas:* 100% (not further specified)	MDR infection: 100%	Defined by improved symptoms, improved imaging where relevant and fever reduction	–	72.2% (13/18)^c^
Munita et al., 2017 [19]	Retrospective cohort	Mixed infections: Pneumonia (N = 3); Pyelonephritis (N = 1), Central line-associated BSI (N = 1), Left ventricular assist device infection (N = 1). Secondary bacteremia is BSI	*Pseudomonas:* 100% (not further specified)	–^e^	Clinical success was defined as a composite of in-hospital survival, resolution of signs and symptoms of the infection (as reported by treating physicians), and absence of recurrence of the infection within the admission	–	66.66% (4/6)^d^

*MDR* multi drug resistant

^a^Undefined primary source

^b^Antibiotic resistance reflected here is not bacteremia specific

^c^For 2 patients, the source of bacteremia was unclear between two sources. Each patient had a possible pneumonia source plus either a wound or UTI

^d^One patient was treated with combination therapy

^e^Three patients (2 with pneumonia and 1 with pyelonephritis) were Carbapenem-resistant

A case series of three patients, which did not specify an underlying infection, reported a clinical cure or success rate at the lower end of that observed across the retrospective studies (66.7%, 2/3) [26] and a separate case report evaluating a patient with skin and soft-tissue infection reported clinical cure or success in this patient (100%, 1/1) [25].

#### Mixed/Not specified bacteremia

Six retrospective cohort studies [10–12, 14, 16, 22], two case reports [24, 27], one case series [28], and one case-control study [29] reported on clinical cure or success among patients with mixed (i.e. primary or secondary) and/or unspecified bacteremia (see Table 3). Across the retrospective cohort studies, clinical cure or success rates were similar to those in reported in studies evaluating only secondary bacteremia: 50% [22] to 91.7% (n = 6 to 27 patients) [12]. Two studies provided IPD for patients with specific infection sources [14, 22] (see Additional File 5). In the first study, all three patients with a respiratory tract infection reported clinical cure or success (100%, 3/3), as did a single patient each with abdominal infection (50%, 1/2), biliary infection (100%, 1/1), and otitis and mastoiditis (100%, 1/1) [14] (see Additional File 5). In the second study, clinical cure or success was achieved by a single patient with septic shock due to cholangitis (100%, 1/1), as well as a patient with combined UTI and deep surgical-site infection (100%, 1/1); IPD were not reported for four other patients with mixed and/or unspecified bacteremia [22] (see Additional File 5).

**Table 3:** Clinical cure or success in mixed/unspecified bacteremia patients receiving C/T

Author, year	Study design	Source	Pathogen type	Antibiotic resistance	Outcome definition	Time point	% (n/N) Reporting clinical cure or success
Case report and case series							
Pezzi et al., 2019 [24]	Case report	Maternal sepsis (not further specified)	*E. coli*: 100%, *K. pneumonia*: 100%^a^	–	–	–	100% (1/1)
Jones et al., 2020 [27]	Case report	–	*Pseudomonas*: 100% (not further specified), *E. coli*: 100%	–	Defined as symptom resolution at the end of therapy, which was defined as documented subjective patient report of no complaints, distress, or disease-specific signs and/ or symptoms at follow-up outpatient physician clinic visits	–	100% (1/1)
Sacha et al., 2017 [28]	Case series	Mixed infections: pneumonia, intra-abdominal, skin and soft tissue, primary bacteremia, bone and joint infection, pleural space infections	–	MDR infection: 40.4%, XDR infection: 25%, PDR infection: NR	–	–	77.8% (7/12)^b^
Case-control study							
Fernandez-Cruz et al., 2019 [29]	Case–control study	Bacteremia secondary n = 7, and primary n = 3	*Pseudomonas:* 100% (not further specified)	MDR infection: 50%, XDR infection: 50%	–	14 days	All patients: 80% (8/10)^c,d^ Monotherapy: 75.0% (3/4)^c^ Combination: 83.3% (5/6)^c^
Retrospective cohort studies							
Bassetti et al., 2019 [12]	Retrospective cohort	Mixed infections: nosocomial pneumonia, ABSSSI, cIAI, cUTI, bone infection and *sepsis*	*Pseudomonas:* 100% (not further specified)	–	Clinical cure or success was defined as complete resolution of clinical signs and symptoms related to P. aeruginosa infection and lack of microbiological evidence of infection	–	70.4% (19/27)
Bassetti et al., 2019 [12]	Retrospective cohort	Mixed infections: nosocomial pneumonia, ABSSSI, cIAI, cUTI, bone infection and *septic shock*	*Pseudomonas:* 100% (not further specified)	–	Clinical cure or success was defined as complete resolution of clinical signs and symptoms related to P. aeruginosa infection and lack of microbiological evidence of infection	–	91.7% (11/12)
Caston et al., 2017 [14]	Retrospective cohort	Overall mixed infections and sepsis/septic shock Mixed hospital-acquired infections: Abdominal (N = 3), Respiratory (N = 6), Otitis and mastoiditis (N = 1), Biliary (N = 1), Venous Central Catheter (N = 1)	*Pseudomonas:* 100% (not further specified)	MDR infection: 100%	Clinical outcome considered a "cure" when attending physician observed a resolution of signs and symptoms and there were no radiologic findings of infection	30 days (after isolation of *P. aeruginosa*)	75.0% (9/12)
Escola-Verge et al., 2018 [16]	Retrospective cohort	Mixed infections: lower respiratory tract, Soft tissue, Urinary tract, Bone, Intra-abdominal, BSI, Mediastinitis	*Pseudomonas*: 100% (not further specified), *Enterobacter*: 13.15%^e^	XDR infection: 100%^f^	Defined as resolution of signs and symptoms of the index infection at 90 days of follow-up	90 days	72.73% (8/11)
Jayakumar et al., 2018 [10]	Retrospective cohort	Mixed infections: Respiratory, Blood, Urinary, Tissue, Wound (patients could have more than one infection)	*Pseudomonas*: 95% (not further specified) • Polymicrobial *Pseudomonas* infection: *Enterobacter* (10%), *Acinetobacter* (10%), *Providencia* (5%), • *Meningosepheum* (5%), • *Morganella* (5%), • *Candida* (14%) *K* *Pneumoniae*: 5%	MDR infection: 86%, all patients with *Pseudomonas infections*)	Documented microbiological eradication or discharge within 30 days from last C/T dose without death	30 days	77.0% (17/22)
						30 days	57.0% (4/7)^g^
						30 days	87.0% (13/15)^h^
King et al., 2018 [11]	Retrospective cohort	Overall Mixed infections: pneumonia, UTI, intra-abdominal, wound	*Pseudomonas:* 100% (not further specified)	MDR infection: 100%	Defined by improved symptoms, improved imaging where relevant and fever reduction	–	76.0% (19/25)
Xipell et al., 2018 [22]	Retrospective cohort	Overall Mixed infections: submandibular fasciitis or UTI and deep surgical-site infection	–	MDR infection: 17.39%, XDR infection: 79%, PDR infection: 4%	–	–	50.0% (3/6)^i^

*ABSSSI* Acute Bacterial Skin and Skin-structure infection, *MDR* Multi drug resistant, *PDR* Pan drug resistant, *XDR* Extensively drug resistant

^a^The presence of candida glabrata in the rectal pad was also found

^b^Authors note that 3 patients have primary bacteremia, 12 patients have concomitant bacteremia but then report outcomes on 12 patients with primary or concomitant bacteremia

^c^There were 10 cases (8, 80% achieved cure), 6 received combination therapy (5, 83.3% achieved cure), 4 received monotherapy (3, 75% achieved cure)

^d^Cases, combination or monotherapy, Combination therapy, 36.4% (12/10) (discrepancy in n/N from publication)

^e^3 Enterococcus faecium, 2 Enterococcus faecalis

^f^Previous XDR-PA isolation 18 (47.4)

^g^Other clinical success

^h^Respiratory clinical success

^i^N represents patients with either confirmed bacteremia, septic shock, or positive blood culture

A case-control study evaluating clinical cure or success in patients with either primary or secondary bacteremia reported a clinical cure or success rate that fell at the higher end of the range reported across the retrospective studies (80%, 8/10) [29]. In this study, similar clinical cure or success rates were reported for patients receiving C/T in combination (83.3%, 5/6) with either amikacin plus levofloxacin (n = 2), amikacin (n = 4), colistin (n = 1), or fosfomycin (n = 1), and those receiving C/T monotherapy (75%, 3/4). Two case reports, neither specifying an underlying infection, reported clinical cure or success in a single patient with maternal sepsis [24] and a single patient with a positive blood culture [27]. A case series also evaluated clinical cure or success in patients with mixed underlying infections (including primary bacteremia); the outcome was achieved by 77.8% (7/12) of patient.

### Microbiological cure or eradication

Eight studies reported microbiological cure or eradication rates among patients with either primary (k = 3 studies) [11, 15, 17, 33], secondary (k = 4) [11, 14, 17, 32] or mixed/unspecified bacteremia (k = 5) [10, 11, 14, 22, 27]. Seven of these provided a definition, all of which relied on a negative culture to measure the outcome. Only one study specified the source of culture (blood) [32]. Three studies specified that a repeat culture was used to measure the outcome [10, 22, 27]. Notably, one study assumed that microbiological cure or eradication was achieved in surviving patients with clinical success, who otherwise did not have a repeat culture available [17]. Despite only few studies specifying that a repeat culture was required, based on clinical practice it is possible that all studies specifying that microbiological cure or eradication was collected, did in fact require a repeat culture.

#### Primary bacteremia

Three retrospective cohort studies reported on microbiological cure or eradication among patients with primary bacteremia and all reported a 100% rate (n = 4 to 7 patients) (see Table 4) [11, 15, 17].

**Table 4:** Microbiological cure or eradication in primary bacteremia patients receiving C/T

Author, year	Study design	Pathogen type	Antibiotic resistance	Outcome definition	Time point	% (n/N) Reporting cure or eradication
Retrospective cohort studies						
Elabor et al., 2018 [15]	Retrospective cohort	*Pseudomonas:* 100% (not further specified)	MDR infection: 100%	Defined as the presence of a repeat negative culture after initiation of treatment	–	100% (4/4)
Gallagher et al., 2018 [17]	Retrospective cohort	*Pseudomonas:* 100% (not further specified)	MDR infection: 100%	Defined as a negative culture at the end of therapy	–	100% (6/6)
King et al., 2018 [11]	Retrospective cohort	*Pseudomonas:* 100% (not further specified)	MDR infection: 100%	Microbiological success required a negative culture at the end of therapy	–	100% (7/7)

*MDR* Multi drug resistant

#### Secondary bacteremia

Three retrospective cohort studies [11, 14, 17] and one single-arm trial [32] reported microbiological cure or eradication to range from 68% [17] to 80% [14] (n = 5 to 9 patients) in patients with secondary bacteremia caused by mixed underlying infections (see Table 5). Two of these studies reported microbiological cure or eradication rates stratified by infection source [11, 14] (see Additional File 5). In a single study providing IPD [14], microbiological cure or eradication was achieved by 66.7% (2/3) of patients with a respiratory tract infection and a single patient each abdominal (100%, 1/1) or venous catheter infection 100% (1/1) (see Additional File 5). In the study providing data for subgroups of patients with specific infections, the microbiological cure or eradication rate was lower among patients with wound infection (0%, 1/1) and pneumonia (38%, 3/8) compared with patients with UTI (86%,6/7) and intra-abdominal infection (100%,1/1) [11] **(**see Additional File 5). A single-arm trial also evaluated microbiological cure or eradication in patients with uncomplicated pyelonephritis or cUTI receiving C/T and reported a rate higher than that observed across all the retrospective cohort studies (95.7%, 22/23) [32].

**Table 5:** Microbiological Cure or Eradication in Secondary Bacteremia Patients Receiving C/T

Author, year	Study design	Source	Pathogen type	Antibiotic resistance	Outcome definition	Time point	% (n/N) Reporting cure or eradication
Single-arm trial							
Arakawa et al., 2019 [32]	Single-arm trial	Mixed infections: uncomplicated pyelonephritis and cUTI	–	–	Negative blood culture	14 days	95.7% (22/23)
Arakawa et al., 2019 [32]	Single-arm trial	Mixed infections: uncomplicated pyelonephritis and cUTI	–	–	Negative blood culture and a urine culture shows all uropathogens (> = 10^5 CFU/ml) found at baseline were decreased to < 10^4 CFU/ml	14 days	78.3% (18/23)
Retrospective cohort studies							
Caston et al., 2017 [14]	Retrospective cohort	Overall mixed infection and bacteremia Mixed infections: abdominal (N = 1), respiratory (N = 3) and venous central catheter (N = 1)	*Pseudomonas:* 100% (not further specified)	MDR infection: 100%	–	30 days (after therapy with C/T)	80.0% (4/5)
Gallagher et al., 2018 [17]	Retrospective cohort	Mixed, bone/ joint, intra-abdominal, pneumonia, wound, and UTI	*Pseudomonas:* 100% (not further specified)	MDR infection: 100%	Defined as a negative culture at the end of therapy	–	68.0% (13/19)
King et al., 2018 [11]	Retrospective cohort	Overall Mixed infections: pneumonia (N = 8), UTI (N = 7), intra-abdominal (N = 4), wound (N = 1)	*Pseudomonas:* 100% (not further specified)	MDR infection: 100%	Microbiological success required a negative culture at the end of therapy	–	72.2% (13/18)^a^

*MDR* Multi drug resistant, *UTI* Urinary tract infections

^a^For 2 patients, the source of bacteremia was unclear between two sources. Each patient had a possible pneumonia source plus either a wound or UTI

#### Mixed/Unspecified bacteremia

Four retrospective cohort studies [10, 11, 14, 22] and one case study [27] reported microbiological cure or eradication in patients with mixed (i.e. primary or secondary) or unspecified bacteremia, which ranged from 60% [22] to 80% (n = 5 to 25 patients) (see Table 6) [11, 14]. Two studies reported data for subgroups of patients with specific infection sources [14, 22] (see Additional File 5). The first study provided IPD and reported the lower rates of microbiological cure or eradication in patients with abdominal (50%, 1/2) and respiratory infection (50%, 1/2) compared with patients with biliary infection (100%, 1/1) or otitis and mastoiditis (100%, 1/1) [14] (see Additional File 5). In the second study, microbiological cure or eradication rate was not achieved by the single patient with submandibular fasciitis (0%, 0/1), but was achieved by the single patient with combined UTI and deep surgical-site infection (100%, 1/1) [22] (see Additional File 5).

**Table 6:** Microbiological cure or eradication in mixed/unspecified bacteremia patients receiving C/T

Author, year	Study design	Source	Pathogen type	Antibiotic resistance	Outcome definition	Time point	% (n/N) Reporting cure or eradication
Case report							
Jones et al., 2020 [27]	Case report	–	*Pseudomonas*: 100% (not further specified), E. coli: 100%	–	Had a clinically evaluable repeat culture demonstrating microbiologic resolution	–	100% (1/1)
Retrospective cohort studies							
Caston et al., 2017 [14]	Retrospective cohort	Overall mixed infections and sepsis/septic shock Mixed hospital-acquired infections: Abdominal, Respiratory, Otitis and mastoiditis, Biliary,	*Pseudomonas:* 100% (not further specified)	MDR infection: 100%	–	30 days (after therapy with C/T)	80.0% (4/5)
Jayakumar et al., 2018 [10]	Retrospective cohort	Mixed infections: Respiratory, Blood, Urinary, Tissue, Wound (patients could have more than one infection)	*Pseudomonas*: 95% (not further specified) • Polymicrobial *Pseudomonas* infection: Enterobacter (10%), Acinetobacter (10%), Providencia (5%), • Meningosepheum (5%), • Morganella (5%), • Candida (14%) *K* *Pneumoniae*: 5%	MDR infection: 86% (all patients with *Pseudomonas infections*)	Documented negative culture of same pathogen after a previously positive culture	–	75.0% (9/12)
King et al., 2018 [11]	Retrospective cohort	Overall Mixed infections: primary bacteremia or pneumonia, UTI, intra-abdominal, wound tested via positive blood culture	*Pseudomonas:* 100% (not further specified)	MDR infection: 100%	Microbiological success required a negative culture at the end of therapy	–	80.0% (20/25)
Xipell et al., 2018 [22]	Retrospective cohort	Overall Mixed infections: submandibular fasciitis or UTI and deep surgical-site infection	–	MDR infection: 17.39%, XDR infection: 79%, PDR infection: 4%	Defined as negative cultures for P. aeruginosa after 72 h of therapy when repeated cultures from the same source were available	–	60.0% (3/5)^a^

*MDR* Multi drug resistant, *PDR* Pan drug resistant, *XDR* Extensively drug resistant

^a^1/3 patients reporting microbiological eradication subsequently developed septic shock and died

### Mortality

Thirteen studies reported mortality rate among patients with either primary (k = 4) [11, 15, 17, 18, 33], secondary (k = 7) [11, 13, 17, 18, 21, 26, 30] or mixed/unspecified bacteremia (k = 7) [10, 11, 14, 16, 21, 22, 29]. Only two reported infection related mortality. Of the remaining 11 studies, one reported that patients had died in hospital, and another the number of patients surviving at study end; the other nine studies did not further define mortality. Mortality was also reported at various time points including 28 days (k = 1) [30], 30 days (k = 8) [10, 11, 13, 15, 17, 21, 26, 29], and 90 days (k = 1) [16]; a single study timed the outcome at 30 days for one patient and at 90 days for another patient [18].

#### Primary bacteremia

Four retrospective cohort studies [11, 15, 17, 18] reported mortality among patients with primary bacteremia, which ranged from 0% [15, 17, 18] to 14% [11] (n = 1 to 7 patients) over a follow-up period of ranging from 30 to 90 days (see Table 7). Three of these four studies reported zero deaths [15, 17, 18], one of which specifically sought to record death due to infection [18].

**Table 7:** Mortality in primary bacteremia patients receiving C/T

Author, year	Study design	Pathogen type	Antibiotic resistance	Outcome definition	Time point	% (n/N) Reporting mortality
Retrospective cohort studies						
Elabor et al., 2018 [15]	Retrospective cohort	*Pseudomonas:* 100% (not further specified)	MDR infection: 100%	–	30 days	0% (0/4)^a^
Gallagher et al., 2018 [17]	Retrospective cohort	*Pseudomonas:* 100% (not further specified)	MDR infection: 100%	Defined as patients who died in the hospital after 30 days	30 days	0% (0/6)
Haidar et al., 2017 [18]	Retrospective cohort (single patient)	*P. aeruginosa:* 100%	MDR infection: 100%	Defined as P. aeruginosa if the patient died with signs and symptoms of infection, microbiologic or histological evidence of an active P. aeruginosa infection, and if other potential causes of death were reasonably excluded	90 days	0% (0/1)^b^
King et al. et al., 2018 [11]	Retrospective cohort	*Pseudomonas:* 100% (not further specified)	MDR infection: 100%	In-hospital mortality	–	0% (0/7)^c^
King et al. et al., 2018 [11]	Retrospective cohort	*Pseudomonas:* 100% (not further specified)	MDR infection: 100%	–	30 days	14.0% (1/7)

MDR, Multi drug resistant

^a^Mortality not explicitly reported, but all 4 patients were reported to survive. This value may need to be evaluated with caution when considering timings for evaluating in-hospital mortality (NR for bacteremia patients). For the overall group of patients, 17/65 (26.1%) of patients died in hospital, yet 56/65 (86.1%) of patients survived to 30 days (indicating that only 9 patients died)

^b^Died within 90 days (Not attributable)

^c^In-hospital mortality

#### Secondary bacteremia

Five retrospective cohort studies [11, 13, 17, 18, 21], an RCT [30] and a case series [26] also reported mortality in patients with secondary bacteremia (see Table 8). Across these studies, mortality ranged vastly from 0% [18] to 100% [13] over a follow-up period ranging from 28 to 30 days (n = 1 to 19 patients).

**Table 8:** Mortality in secondary bacteremia patients receiving C/T

Author, year	Study design	Source	Pathogen type	Antibiotic resistance	Outcome definition	Time point	% (n/N) Reporting mortality
Case series							
Hakki and Lewis et al., 2018 [26]	Case series	NR^a^	*P. aeruginosa:* 100%	MDR infection: 100%	Defined as patients surviving	30 days	0% (0/3)
Randomized controlled trials (RCT)							
Kollef et al. et al., 2019 [30]	RCT	Nosocomial pneumonia	*Pseudomonas*^b^: 17.4% (Not further specified), *Enterobacter:* 53.86%^c^	–	–	28 days	52.0% (13/25)
Retrospective cohort studies							
Bosaeed et al., 2020 [13]	Retrospective cohort (single patient)	Complicated perianal abscesses	*Pseudomonas:* 100% (not further specified)	MDR infection: 100%	–	30 days	100% (1/1)
Gallagher et al., 2018 [17]	Retrospective cohort	Mixed infections: bone/ joint, intra-abdominal, pneumonia, wound, and UTI	*Pseudomonas:* 100% (not further specified)	MDR infection: 100%	–	–	36.8% (7/19)
Haidar et al., 2017 [18]	Retrospective cohort	Pneumonia	*P. aeruginosa:* 100%	MDR infection: 100%^c^	Defined as P. aeruginosa if the patient died with signs and symptoms of infection, microbiologic or histological evidence of an active P. aeruginosa infection, and if other potential causes of death were reasonably excluded	30 days for patient 1 90 days for patient 2	0% (0/2)
King et al., 2018 [11]	Retrospective cohort	Mixed infections: Pneumonia (N = 8), UTI (N = 7), intra-abdominal (N = 4), wound (N = 1)	*Pseudomonas:* 100% (not further specified)	MDR infection: 100%	–	30 days	44.4% (8/18)^d,e,f^
Rodriguez-Nunez et al., 2019 [21]	Retrospective cohort	Lower respiratory tract infection	*Pseudomonas:* 100% (not further specified)	–^g^	–	30 days	25.0% (1/4)

*MDR* Multi drug resistant

^a^Undefined primary source

^b^Calculated % tallying number of patients with Pseudomonas pathogen

^c^Antibiotic resistance presented here not specific for bacteremia

^d^Died within 30 days (not attributable)

^e^In-hospital mortality

^f^For 2 patients, the source of bacteremia was unclear between two sources. Each patient had a possible pneumonia source plus either a wound or UTI

^g^Multi-drug resistant or extensive-drug resistant, exact resistance measure unclear

Mortality differed by underlying infection source and was also wide-ranging for the same infection evaluated across multiple studies. A single retrospective cohort study reported mortality stratified by infection source [11] (see Additional File 5). Reported at 30 days, mortality was higher in patients with wound infection (100%, 1/1) and pneumonia (63%, 5/8) compared with UTI (29%, 2/7) and intra-abdominal infection (0%, 0/4) [11]. Apart from this study, three others also reported on patients with respiratory tract infections; in an RCT 52% (13/25) [30] of nosocomial pneumonia patients died at 28 days, in a retrospective cohort study 25% (1/4) [21] of patients with lower respiratory tract infection died at 30 days, and in another retrospective cohort study 0% (1/2) of pneumonia patients died at 30 days [18] (see Additional File 5).

#### Mixed/Unspecified bacteremia

Six retrospective cohort studies [10, 11, 14, 16, 21, 22] and one case–control study [29] reported mortality in patients with either mixed (i.e. primary or secondary) or unspecified bacteremia (see Table 9). Across these data mortality at various assessment points ranged from 0% to 51.6% (n = 2 to 19 patients); for studies reporting time points, all but one reporting a follow-up period of 90 days (mortality: 36.4%)[16], evaluated mortality at 30 days. Three studies fell at the upper end of this range; the first evaluated only patients with lower respiratory tract infections (51.6%, 16/31) [21] and the other two evaluated a higher proportion of patients with XDR infection (79%[22]-100% [16] versus 0%). Removing these studies, mortality ranged from 10%[10] to 28% [11]. Across all studies, only one specified that death was due to infection [16].

**Table 9:** Mortality in mixed/unspecified bacteremia patients receiving C/T

Author, year	Study design	Source	Pathogen type	Antibiotic resistance	Outcome definition	Time point	% (n/N) Reporting mortality
Case–control study							
Fernandez-Cruz et al., 2019 [29]	Case–control study	Bacteremia secondary n = 7, and primary n = 3	*Pseudomonas:* 100% (not further specified)	MDR infection: 50%, XDR infection: 50%	–	30 days	All patients: 10.0% (1/10)^a, b^ Combination therapy: 25.0% (1/4)^a,^ Monotherapy: 0% (0/6)^a^
Retrospective cohort studies							
Caston et al., 2017 [14]	Retrospective cohort	Mixed hospital-acquired infections: Abdominal, Respiratory, Otitis and mastoiditis, Biliary, Venous Central Catheter	*Pseudomonas:* 100% (not further specified)	MDR infection: 100%	–	–	25.0% (3/12)
Escola-Verge et al., 2018 [16]	Retrospective cohort	Mixed infections: lower respiratory tract, Soft tissue, Urinary tract, Bone, Intra-abdominal, BSI, Mediastinitis	*Pseudomonas:* 100% (not further specified)	XDR infection: 100%	Defined as the composite endpoint of attributable mortality, or a persistent or recurrent XDR-PA index infection at 90 days of follow-up. In accordance with previous studies, mortality was attributed to XDR-PA infection if the patient had signs and symptoms of infection at death or microbiologic evidence of an active XDR-PA infection, and other potential causes of death had been excluded	90 days	36.4% (4/11)^c^
Jayakumar et al., 2018 [10]	Retrospective cohort	Mixed infections: Respiratory, Blood, Urinary, Tissue, Wound (patients could have more than one infection)	*Pseudomonas:* 95% (not further specified), *K. pneumoniae*: 5%, Polymicrobial infection^e^: *Enterobacter* (10%), *Acinetobacter* (10%), *Providencia* (5%), *Meningosepheum* (5%), *Morganella* (5%), *Candida* (14%)	MDR infection: 86%	–	30 days	10.0% (2/20)^d^
					–	30 days	24.0% (5/21)
King et al., 2018 [11]	Retrospective cohort	Mixed infections: pneumonia, UTI, intra-abdominal, wound	*Pseudomonas:* 100% (not further specified)	MDR infection: 100%	–	–	24.0% (6/25)^e^
					–	30 days	28.0% (7/25)
Rodriguez-Nunez et al., 2019 [21]	Retrospective cohort	Lower respiratory tract infection	*Pseudomonas:* 100% (not further specified)	–^f^	–	30 days	51.6% (16/31)
Xipell et al., 2018 [22]	Retrospective cohort	Mixed infections: submandibular fasciitis or UTI and deep surgical-site infection	–	MDR infection: 17.39%,XDR infection: 79%, PDR infection: 4%^g^	–	–	50.0% (3/6)^h^

*MDR* Multi drug resistant, *PDR* Pan drug resistant, *XDR* Extensively drug resistant

^a^There were 10 cases (8, 80% achieved cure), 6 received combination therapy (5, 83.3% achieved cure), 4 received monotherapy (3, 75% achieved cure)

^b^Cases, combination or monotherapy, Combination therapy, 36.4% (12/10) (discrepancy in n/N from publication)

^c^Table 2 of the study PDF reports 3/12 patients reporting clinical failure had a positive blood culture. The text also notes that 4/12 clinical failures are due to death by sepsis

^d^Infection-related mortality

^e^In-hospital mortality

^f^Multi-drug resistant or extensive-drug resistant, exact resistance measure unclear

^g^Poly-microbial infection

^h^N represents patients with either confirmed bacteremia, septic shoc

### Other efficacy measures

The SLR also sought to capture data on composite clinical response, relapse, hospital re-admission, and hospital length of stay, which were rarely reported in the literature. Only a single study each reported on these outcomes. In general, it was found that treatment with C/T led to favorable results with respect to these measures.

In an RCT evaluating patients with bacteremia secondary to pyelonephritis or complicated lower UTI, a composite clinical response rate (defined as clinical cure and microbiological eradication of all baseline pathogens) of 79.3% was reported. The study did not report on primary pathogen type, antibiotic resistance status, or time point [31]. In a retrospective cohort study of patients with either primary bacteremia (n = 1) or bacteremia secondary to MDR *Pseudomonas* pneumonia (n = 2), no patient reported relapse or recurrent infection (outcomes not defined, reported at 90 days) [18]. In addition to C/T, the primary bacteremia patient received ciprofloxacin and inhaled tobramycin, one secondary bacteremia patient received gentamicin; the third patient was treated with C/T monotherapy [18]. In another retrospective cohort study of patients with mixed or unspecified bacteremia and mixed antibiotic resistance, the need for hospital readmission was rare; at 30 days 14% of patients were admitted to the hospital from which 10% were infection-related re-admissions [10]. The final retrospective cohort study reported hospital length of stay among patients with secondary bacteremia due to either MDR or XDR *Pseudomonas* infections. Median length of hospital stay from the onset of bacteremia was 14.5 (IQR: 9 to 26) days [20].

## Discussion

Bacteremia causes a considerable disease burden globally, with a substantial impact both in terms of patient morbidity and economic impact [2, 3]. This review sought to provide a summary of the available literature on C/T, a novel antibacterial agent, as utilized in patients with primary, secondary, mixed or unspecified bacteremia. Limited data and substantial variability in patient characteristics across studies make it difficult to draw any substantial conclusions, but the overall results were favorable to C/T.

The 23 studies included in the review provided mostly observational evidence (i.e. cohort and case-control studies, case reports, case series), though few interventional studies were included (i.e. RCTs and single-arm trials). The majority evaluated patients with *Pseudomonas* infections (range: 95% to 100% of patients in 20/23 included studies), which are of particular concern as they are known to cause severe infections and to exhibit both intrinsic and acquired resistance to antibiotics [12, 34]. Careful selection of an appropriate therapy is critical when dealing with antibiotic resistant bacteria, a step which may lead to delays in treatment. Agents commonly used to treat these infections include aminoglycosides, β-lactams, fluoroquinolones, and/or polymyxins but a growing evidence base suggests increasing resistance to these treatments [23, 34]. The need for newer effective agents or combinations of existing therapies is apparent. Recently, the challenges involved in treating highly resistant bacteria has led the World Health Organization (WHO) to identify a crucial need for effective antibiotic treatment of resistant *P. aeruginosa* infections [9, 34].

C/T presents as a potentially promising therapeutic option. In most studies identified by the review (16/23), treatment of bacteremia was complicated by the frequency of MDR, PDR, and XDR infections. C/T generally led to favorable clinical efficacy based on reported clinical cure or success, microbiological cure or success, and mortality. Few data points suggest that patients with XDR infections exhibited worse outcomes than those with MDR when considering clinical cure or success [22, 23], though this trend was not observed for either microbiological cure or eradication, or, mortality [16, 29]. Across the evidence base, patients with respiratory infections also exhibited worse outcomes than those with other underlying infection sources, reporting lower rates of clinical cure or success, lower rates of microbiological cure or eradication, as well as higher mortality compared with other infection types calling attention to the difficulty in treating respiratory infections and the underlying critical nature of patients with secondary bacteremia. Notably, the studies evaluating patients with mixed infection types did not report on the dose of C/T that was administered to patients with respiratory infections. Dosing of C/T used for respiratory infections is double the recommended dose used for cUTI and cIAI, which had earlier regulatory approval. It is possible that potential underdosing of patients with respiratory infections may have contributed to the poorer prognosis seen in patients with pneumonia. The need for ventilators and presence of a greater number of comorbid conditions may also have contributed to the lower cure rates and higher mortality among patients with respiratory infections [17].

The observed trends related to XDR and respiratory infections are aligned with what is expected in these patient populations [35]. While inconsistency in some of the findings of the review is likely due to the inclusion of studies with small sample sizes and heterogeneity in key patient characteristics, interpretation of data identified by the review is subject to additional challenges. A key limitation of this review is that most included studies did not assess bacteremia outcomes as a primary aim of the study. The presence of bacteremia was reported either, clearly, as part of study eligibility criteria or patient baseline characteristics or was inferred based on reporting of positive blood culture, sepsis, or septic shock. Often, outcomes data for bacteremia patients were not separately reported and had to be hand calculated based on details provided by authors in provided text, tables, and figures. This reiterates the separate focus of the studies from which the bacteremia population was identified. While a number of the included studies specified whether patients with primary or secondary bacteremia were evaluated, several reported on either a mixed (for which primary or secondary bacteremia results were not separable), or an unspecified bacteremia population. Some studies also reported conflicting data, which are captured in footnotes to our results tables. All included studies were of small sample size and even larger studies of patients with mixed infections reported on few patients with bacteremia (n = 1 to 31). As previously mentioned, there was much heterogeneity in data evaluated and reported in relation to presence of primary or secondary bacteremia, pathogen type, antibiotic resistance, underlying infection, C/T dose and outcome definitions. Many studies reported only overall population characteristics as opposed to those specific to the bacteremia cohort, and thus did not accurately represent the subset of patients with bacteremia. Further, most evidence was collected from observational studies or case reports, adding another layer of variability, particularly related to treatment characteristics. The patients included in these studies may have experienced varied durations of treatment, times to appropriate treatment, use of combination therapy, source control and dosing, which may also explain wide ranges of reported outcomes and inconsistency in any observed trends even when patients with the same type of bacteremia and resistance levels were evaluated. Finally, although the SLR was conducted in accordance with established guidance, as always reviews are limited by the confines of their selection criteria and the risk of publication bias present within the included evidence base.

## Conclusion

Although the available evidence and observed trends for C/T in bacteremia should be interpreted with caution, the direction of effect would support the utilization of C/T for these difficult to treat infections. Treatment with C/T was generally found to lead to favorable clinical efficacy based on reported clinical cure or success, microbiological cure or success, and mortality among patients with bacteremia often caused by MDR/PDR/XDR *Pseudomonas* infections. Future research should aim to supplement the published data by conducting larger studies that use methods most often implemented in the existing evidence base and also consider the impact of treatment effect modifiers, such as bacteremia source (i.e., pathogen type, antibiotic resistance, underlying infection), infection severity, comorbidities, and treatment regimen, as well as providing outcome data based on key parameters such as presence of bacteremia, pathogen and resistance profile.

## Supplementary Information

**Additional file 1.** Selection criteria based on the Population, Interventions, Comparisons, Outcomes, Time, and Study design (PICOTS) structure.

**Additional file 2.** Literature search strategies. Embase (1974 to February 16, 2020; Search executed: February 17, 2020). Medline (Ovid MEDLINE(R) In-Process & Other Non-Indexed Citations, Ovid MEDLINE(R) Daily and Ovid MEDLINE(R) 1946 to February 16, 2020; Search executed: February 17, 2020). CCTR (EBM Reviews - Cochrane Central Register of Controlled Trials January 2020; Search executed: February 17, 2020).

**Additional file 3.** PRISMA diagram.

**Additional file 4.** Reported outcome definitions. Reported Definitions of Clinical Cure or Success. Reported Definitions of Microbiological Cure or Eradication. Reported Definitions of Mortality.

**Additional file 5.** Infection-specific individual patient data, where available. Clinical Cure or Success (Secondary Bacteremia), Reported Infection-specific Individual Patient Data. Clinical Cure or Success (Mixed/Unspecified Bacteremia), Reported Infection-specific Individual Patient Data. Microbiological Cure or Eradication (Secondary Bacteremia), Reported Infection-specific Individual Patient Data. Microbiological Cure or Eradication (Mixed/Unspecified Bacteremia), Reported Infection-specific Individual Patient Data. Mortality (Secondary Bacteremia), Reported Infection-specific Individual Patient Data. Mortality (Mixed/Unspecified Bacteremia), Reported Infection-specific Individual Patient Data.

## Data Availability

Key data generated during the study and analyzed within this manuscript are included in the tables within this article and its supplementary files.

## Abbreviations

BSI: Bloodstream infection
C/T: Ceftolozane/tazobactam
cIAI: Complicated intra-abdominal infections
cUTI: Complicated urinary tract infection
ECCMID: European congress of clinical microbiological and infectious diseases
EMA: European medicines agency
FDA: Food and drug administration (US)
HABP: Hospital-acquired bacterial pneumonia
k: Number of studies
MDR: Multi-drug resistant
MODS: Multiple organ dysfunction syndrome
n: Number of patients
PDR: Pan-drug resistant
PICOTS: Population, interventions, comparisons, outcomes, time and study design
PRISMA: Preferred reporting items for systematic reviews and meta-analyses
RCT: Randomized controlled trial
SIRS: Systemic inflammatory response syndrome
SLR: Systematic literature review
US: United States
UTI: Urinary tract infection
VABP: Ventilator-associated bacterial pneumonia
VNP: Ventricular-associated nosocomial pneumonia
WHO: World Health Organization
XDR: Extensively drug resistant
