# Supplementary material for: Ceftolozane/tazobactam for the treatment of bacteremia: a systematic literature review (SLR)
Source: Ann Clin Microbiol Antimicrob. 2022 Oct 3;21:42. doi: 10.1186/s12941-022-00528-0 (PMC9531517; doi:10.1186/s12941-022-00528-0)
Supplement: Supplementary file 1 — Additional file 1. Selection criteria based on the Population, Interventions, Comparisons, Outcomes, Time, and Study design (PICOTS) structure. [file 12941_2022_528_MOESM1_ESM.docx]

Additional File 1: Selection criteria based on the Population, Interventions, Comparisons, Outcomes, Time and Study design (PICOTS) structure

Study selection criteria (PICOTS)

|  | Inclusion Criteria |
| --- | --- |
| Population(s) | Adult patients with (≥18 years) with primary or secondary bacteremia*  **Studies of mixed infection populations, which separately report outcomes of interest for a bacteremia population will also be included.* |
| Interventions | C/T (Zerbaxa) |
| Comparisons | - Any of the interventions of interest - Other active treatments - Best Supportive Care (BSC) - Standard of Care (SOC) - Placebo |
| Outcomes | - Up to 12 clinical outcomes, including but not limited to: - Clinical cure or success - Microbiological cure or eradication - Composite cure - Mortality - Hospital length of stay - Intensive care unit (ICU) length of stay - Hospital re-admission (30 days, 60 days, 90 days) - Relapse (30 days, 60 days, 90 days) |
| Time | No limit applied to search strategy |
| Study design | - Observational studies (e.g., prospective and retrospective cohort studies, case-control studies, cross-sectional studies, time-in-motion studies, controlled and uncontrolled longitudinal studies) - Single arm studies - Case series/ studies - Randomized controlled trials and non-randomized clinical trials |
| Other | Included studies will be reported in English language only  No limitation will be applied based on geography |
