# Supplementary material for: Ceftolozane/tazobactam for the treatment of bacteremia: a systematic literature review (SLR)
Source: Ann Clin Microbiol Antimicrob. 2022 Oct 3;21:42. doi: 10.1186/s12941-022-00528-0 (PMC9531517; doi:10.1186/s12941-022-00528-0)
Supplement: Supplementary file 2 — Additional file 2. Literature search strategies. Embase (1974 to February 16, 2020; Search executed: February 17, 2020). Medline (Ovid MEDLINE(R) In-Process & Other Non-Indexed Citations, Ovid MEDLINE(R) Daily and Ovid MEDLINE(R) 1946 to February 16, 2020; Search executed: February 17, 2020). CCTR (EBM Reviews - Cochrane Central Register of Controlled Trials January 2020; Search executed: February 17, 2020). [file 12941_2022_528_MOESM2_ESM.docx]

Additional File 2: Literature search strategies

Embase (1974 to February 16, 2020; Search executed: February 17, 2020)

| **No.** | **Criteria** | **Strings** | **Hits** |
| --- | --- | --- | --- |
| 1 | Intervention | Zerbaxa or (Zerbaxa.mp) | 6 |
| 2 |  | (ceftolozane or 'CXA-201' or 'cb-500,201' OR cb500201 OR 'cxa-201' or cxa201 or 'cxa 101 plus tazobactam' or 'ceftolozane tazobactam' or 'ceftolozane plus tazobactam' or 'ceftolozane-tazobactam' or (ceftolozane adj2 tazobactam) or MK7655-A or MK7655A).mp. | 919 |
| ***3*** | ***Combined Intervention*** | ***1 or 2*** | ***920*** |

Medline (Ovid MEDLINE(R) In-Process & Other Non-Indexed Citations, Ovid MEDLINE(R) Daily and Ovid MEDLINE(R) 1946 to February 16, 2020; Search executed: February 17, 2020)

| **No.** | **Criteria** | **Strings** | **Hits** |
| --- | --- | --- | --- |
| 1 | Intervention | Zerbaxa or (Zerbaxa.mp) | 9 |
| 2 |  | (ceftolozane or 'CXA-201' or 'cb-500,201' OR cb500201 OR 'cxa-201' or cxa201 or 'cxa 101 plus tazobactam' or 'ceftolozane tazobactam' or 'ceftolozane plus tazobactam' or 'ceftolozane-tazobactam' or (ceftolozane adj2 tazobactam) or MK7655-A or MK7655A).mp. | 435 |
| ***3*** | ***Combined Intervention*** | ***1 or 2*** | ***435*** |

CCTR (EBM Reviews - Cochrane Central Register of Controlled Trials January 2020; Search executed: February 17, 2020)

| **No.** | **Criteria** | **Strings** | **Hits** |
| --- | --- | --- | --- |
| 1 | Intervention | Zerbaxa or (Zerbaxa.mp) | 5 |
| 2 |  | (ceftolozane or 'CXA-201' or 'cb-500,201' OR cb500201 OR 'cxa-201' or cxa201 or 'cxa 101 plus tazobactam' or 'ceftolozane tazobactam' or 'ceftolozane plus tazobactam' or 'ceftolozane-tazobactam' or (ceftolozane adj2 tazobactam) or MK7655-A or MK7655A).mp. | 53 |
| ***3*** | ***Combined Intervention*** | ***1 or 2*** | ***53*** |
