# Supplementary material for: Ceftolozane/tazobactam for the treatment of bacteremia: a systematic literature review (SLR)
Source: Ann Clin Microbiol Antimicrob. 2022 Oct 3;21:42. doi: 10.1186/s12941-022-00528-0 (PMC9531517; doi:10.1186/s12941-022-00528-0)
Supplement: Supplementary file 3 — Additional file 3. PRISMA diagram. [file 12941_2022_528_MOESM3_ESM.docx]

Additional File 3: PRISMA diagram

**Screening**

**Included**

**Eligibility**

**Identification**

**Citations identified through database searching**n = 1,455

- Embase (n = 944)
- MEDLINE (n = 452)
- CCTR (n=59)

**Duplicate Citations Removed**

n = 445

**Titles/Abstracts Reviewed**

n = 1,010

**Titles/abstracts excluded**

n = 916

**Full-text articles reviewed**
n = 94

**Full-text articles excluded**

n = 73

- *Population*, no infection of interest (n=40)
- *Outcome*, not separable for infection of interest (n=13)
- *Outcome*, not of interest (i.e. not clinical efficacy) (n=8)
- *Study design*, irrelevant (n=8)
- *Other*, Non-English language (n=2)
- *Intervention*, No Zerbaxa/CT (n=1)
- *Population*, <18 years old (n=1)

Full-text articles included

n = 21

**Included in SLR**

**n = 24 publications; 23 studies**

**Added Material from Grey Literature**

n=3

- 2018 and 2019 conference abstracts from ECCMID or IDWeek (n=3)
