# Supplementary material for: Ceftolozane/tazobactam for the treatment of bacteremia: a systematic literature review (SLR)
Source: Ann Clin Microbiol Antimicrob. 2022 Oct 3;21:42. doi: 10.1186/s12941-022-00528-0 (PMC9531517; doi:10.1186/s12941-022-00528-0)
Supplement: Supplementary file 4 — Additional file 4. Reported outcome definitions. Reported Definitions of Clinical Cure or Success. Reported Definitions of Microbiological Cure or Eradication. Reported Definitions of Mortality. [file 12941_2022_528_MOESM4_ESM.docx]

Additional File 4: Reported outcome definitions

Reported Definitions of Clinical Cure or Success

| **Definition** | **Studies** |
| --- | --- |
| Resolution of signs and symptoms present at diagnosis or baseline | Elabor 2018([15](#_ENREF_15)), Escola-Verge 2018([16](#_ENREF_16)) (90 days), Jones 2020([27](#_ENREF_27)) |
| Improvement or resolution of signs and symptoms from baseline **plus**   - Fever reduction | Gallagher 2018([17](#_ENREF_17)) |
| Improvement or resolution of signs and symptoms from baseline **plus**   - Fever reduction - Improved imaging (details related to imaging not specified) | King 2018([11](#_ENREF_11)), Caston 2017([14](#_ENREF_14)) (30 days after isolation of *Pseudomonas* infection) |
| Resolution of signs and symptoms present at diagnosis or baseline **plus**   - Lack of microbiological evidence of infection | Bassetti 2019([12](#_ENREF_12)) |
| Resolution of signs and symptoms present at diagnosis or baseline **plus**   - No recurrence of infection at same location during admission | Xipell 2018([22](#_ENREF_22)) |
| Resolution of signs and symptoms present at diagnosis or baseline **plus**   - Repeat microbiological clearance - 30-day in-hospital survival after initiation of C/T | Bosaeed 2020([13](#_ENREF_13)) (14 days) |
| Resolution of signs and symptoms present at diagnosis or baseline **plus**   - Clearance of bacteremia   No recurrence (defined as signs, symptoms plus positive culture) | Hakki and Lewis 2018([26](#_ENREF_26)) (30 days) |
| Resolution of signs and symptoms present at diagnosis or baseline **plus**   - In-hospital survival - Absence of recurrence of infection within hospital admission | Munita 2017([19](#_ENREF_19)) |
| Resolution of signs and symptoms present at diagnosis or baseline **plus**   - No new signs, symptoms, or complications attributable to infection (ventricular-associated pneumonia [VNP]) - No additional antibiotic therapy, except for approved adjunctive treatment in surviving patients | Kollef 2019([30](#_ENREF_30)) |
| Repeat microbiological clearance | Jayakumar 2018([10](#_ENREF_10)) (30 days) |
| Derived from patients reporting clinical failure | Haidar 2017([18](#_ENREF_18)), Diaz-Canestro 2018([23](#_ENREF_23)) |

Reported Definitions of Microbiological Cure or Eradication

| **Definition** | **Studies** |
| --- | --- |
| Negative culture, source of culture: not specified | Elabor 2018([15](#_ENREF_15)), King 2018([11](#_ENREF_11)), Gallagher 2018([17](#_ENREF_17)), Jayakumar 2018([10](#_ENREF_10)), Xipell 2018([22](#_ENREF_22)) |
| Negative culture, source of culture: blood  *An additional negative urine culture was used to define “successful outcome” in this study | Arakawa 2019([32](#_ENREF_32)) (14 days) |
| Repeat negative culture demonstrating microbiological resolution, source of culture: not specified | Jones 2020([27](#_ENREF_27)) |

Reported Definitions of Mortality

| **Definition** | **Studies** |
| --- | --- |
| Patient dying with signs and symptoms of infection, microbiological or histological evidence of active infection, and with other potential causes of death reasonably excluded. | Haidar 2017([18](#_ENREF_18)) |
| Patient dying with a persistent or recurrent XDR infection | Escola-Verge 2018([16](#_ENREF_16)) |
| Patients dying in hospital | Gallagher 2018([17](#_ENREF_17)) |
| Derived from patients reported to survive | Hakki and Lewis 2018([26](#_ENREF_26)) |
