# Supplementary material for: Ceftolozane/tazobactam for the treatment of bacteremia: a systematic literature review (SLR)
Source: Ann Clin Microbiol Antimicrob. 2022 Oct 3;21:42. doi: 10.1186/s12941-022-00528-0 (PMC9531517; doi:10.1186/s12941-022-00528-0)
Supplement: Supplementary file 5 — Additional file 5. Infection-specific individual patient data, where available. Clinical Cure or Success (Secondary Bacteremia), Reported Infection-specific Individual Patient Data. Clinical Cure or Success (Mixed/Unspecified Bacteremia), Reported Infection-specific Individual Patient Data. Microbiological Cure or Eradication (Secondary Bacteremia), Reported Infection-specific Individual Patient Data. Microbiological Cure or Eradication (Mixed/Unspecified Bacteremia), Reported Infection-specific Individual Patient Data. Mortality (Secondary Bacteremia), Reported Infection-specific Individual Patient Data. Mortality (Mixed/Unspecified Bacteremia), Reported Infection-specific Individual Patient Data. [file 12941_2022_528_MOESM5_ESM.docx]

Additional File 5: Infection-specific individual patient data, where available

Clinical Cure or Success (Secondary Bacteremia), Reported Infection-specific Individual Patient Data

| **Author, Year** | **Study Design** | **Source** | **Pathogen type** | **Antibiotic resistance** | **Outcome Definition** | **Time Point** | **% (n/N)**  **Reporting Clinical Cure or Success** |
| --- | --- | --- | --- | --- | --- | --- | --- |
| **Case Report and Case Series** | | | | | | | |
| Hakki and Lewis et al, 2018([26](#_ENREF_26)) | Case series | NR | *Pseudomonas:* 100% (not further specified) | MDR infection: 100% | Defined as resolution of signs and symptoms of the infection during treatment with C/T, clearance of bacteremia (if present) within  72 h of initiation of C/T, and absence of infection recurrence, defined as signs and symptoms of infection along with culture positivity for P. aeruginosa while receiving C/T or within 30 days of completion of C/T therapy | 30 days | 100% (1/1) |
|  |  |  |  |  |  |  | 0% (0/1) |
|  |  |  |  |  |  |  | 100% (1/1) |
| **Retrospective cohort studies** | | | | | | | |
| Caston et al, 2017([14](#_ENREF_14)) | Retrospective cohort (single patient) | Venous central catheter | *Pseudomonas:* 100% (not further specified) | MDR infection: 100% | Clinical outcome considered a "cure" when attending physician observed a resolution of signs and symptoms and there were no radiologic findings of infection. | 30 days (after isolation of P. aerginosa) | 100% (1/1) |
|  |  | Respiratory (not further specified) |  |  |  |  | 0% (0/1)^b^ |
|  |  | Respiratory (not further specified) |  |  |  |  | 100% (1/1) |
|  |  | Respiratory (not further specified) |  |  |  |  | 0% (0/1)^b^ |
|  |  | Abdominal (not further specified) |  |  |  |  | 100% (1/1) |
| Haidar et al, 2017([18](#_ENREF_18)) | Retrospective cohort (single patient) | Pneumonia | *P. aeruginosa:* 100% | MDR infection: 100% | Clinical failure was defined as attributable mortality due to P. aeruginosa, persistent signs or symptoms of infection or positive culture despite ≥7 days of C/T, or recurrent P. aeruginosa infection (recurrent signs and symptoms and recurrent culture positivity within 90 days) | -- | 100% (1/1)^c^ |
|  |  | Pneumonia |  |  |  |  | 100% (1/1)^c^ |
| King et al, 2018([11](#_ENREF_11)) | Retrospective cohort | Pneumonia | *Pseudomonas:* 100% (not further specified) | MDR infection: 100% | Defined by improved symptoms, improved imaging where relevant and fever reduction | -- | 38% (3/8) |
|  |  | Urinary tract |  |  |  |  | 86% (6/7) |
|  |  | Intra-abdominal |  |  |  |  | 100% (4/4) |
|  |  | Wound |  |  |  |  | 0% (0/1) |
| Munita et al, 2017([19](#_ENREF_19)) | Retrospective cohort | Pneumonia, bacteremia is reported as BSI | *Pseudomonas:* 100% (not further specified) | --^d^ | Clinical success was defined as a composite of in-hospital survival, resolution of signs and symptoms of the infection (as reported by treating physicians), and absence of recurrence of the infection within the admission | -- | 100% (1/1) |
|  |  | Pyelonephritis, bacteremia is reported as BSI |  | --^d^ |  |  | 100% (1/1) |
|  |  | Central line-associated BSI |  | -- |  |  | 100% (1/1) |
|  |  | Left ventricular assist device infection, bacteremia is reported as BSI |  | -- |  |  | 100% (1/1) |
|  |  | Pneumonia, bacteremia is reported as BSI |  | --^d^ |  |  | 0% (0/1) |
|  |  | Pneumonia, bacteremia is reported as BSI |  | -- |  |  | 0% (0/1) |

***Abbreviations****: MDR, Multi drug resistant*

*a Undefined primary source;*

*b Occurrence of death*

*c An extracted number of 1 denotes clinical success;*

*d Carbapenem-resistant*

Clinical Cure or Success (Mixed/Unspecified Bacteremia), Reported Infection-specific Individual Patient Data

| **Author, Year** | **Study Design** | **Source** | **Pathogen type** | **Antibiotic resistance** | **Outcome Definition** | **Time Point** | **% (n/N)**  **Reporting Clinical Cure or Success** |
| --- | --- | --- | --- | --- | --- | --- | --- |
| **Retrospective cohort studies** | | | | | | | |
| Caston et al, 2017([14](#_ENREF_14)) | Retrospective cohort (single patient) | Otitis and mastoiditis and sepsis | *Pseudomonas:* 100% (not further specified) | MDR infection: 100% | Clinical outcome considered a "cure" when attending physician observed a resolution of signs and symptoms and there were no radiologic findings of infection. | 30 days (after isolation of P. aerginosa) | 100% (1/1)^g^ |
|  |  | Biliary (not further specified) and sepsis/septic shock |  |  |  |  | 100% (1/1) |
|  |  | Abdominal (not further specified) and sepsis/septic shock |  |  |  |  | 0% (0/1)^h^ |
|  |  | Abdominal (not further specified) and sepsis/septic shock |  |  |  |  | 100% (1/1) |
|  |  | Respiratory (not further specified) and sepsis/septic shock |  |  |  |  | 100% (1/1) |
|  |  | Respiratory (not further specified) and severe sepsis |  |  |  |  | 100% (1/1) |
|  |  | Respiratory (not further specified) and sepsis/septic shock |  |  |  |  | 100% (1/1) |
| Xipell et al, 2018([22](#_ENREF_22)) | Retrospective cohort (single patient) | UTI and deep surgical-site infection | *Pseudomonas:* 100% (not further specified), Enterobacter: 100%, K. pneumonia: 100%^n^ | PDR infection: 100% | Defined as resolution of signs and symptoms of the infection (as reported by treating physicians) and no recurrence of the infection in the same location during admission | **--** | 100% (1/1) |
|  |  | Septic shock due to cholangitis | *Pseudomonas*: 100% (not further specified), E. coli: (100%^b^ | XDR infection: 100% |  |  | 100% (1/1) |

***Abbreviations:*** *MDR, Multi drug resistant; PDR, Pan drug resistant; XDR, Extensively drug resistant*

*a Late occurrence;*

*b Poly microbial infection*

Microbiological Cure or Eradication (Secondary Bacteremia), Reported Infection-specific Individual Patient Data

| **Author, Year** | **Study Design** | **Source** | **Pathogen type** | **Antibiotic resistance** | **Outcome Definition** | **Time Point** | **% (n/N)**  **Reporting Cure or Eradication** |
| --- | --- | --- | --- | --- | --- | --- | --- |
| **Retrospective cohort studies** | | | | | | | |
| Caston et al, 2017([14](#_ENREF_14)) | Retrospective cohort (single patient) | Venous central catheter | *Pseudomonas:* 100% (not further specified) | MDR infection: 100% | -- | 30 days (after therapy with C/T) | 100% (1/1) |
|  |  | Respiratory (not further specified) |  |  |  |  | 100% (1/1) |
|  |  | Respiratory (not further specified) |  |  |  |  | 100% (1/1) |
|  |  | Respiratory (not further specified) |  |  |  |  | 0% (0/1)^a^ |
|  |  | Abdominal (not further specified) |  |  |  |  | 100% (1/1) |
| King et al, 2018([11](#_ENREF_11)) | Retrospective cohort | Pneumonia | *Pseudomonas:* 100% (not further specified) | MDR infection: 100% | Microbiological success required a negative culture at the end of therapy | -- | 38% (3/8) |
|  |  | Urinary tract |  | MDR infection: 100% |  |  | 86% (6/7) |
|  |  | Intra-abdominal |  | MDR infection: 100% |  |  | 100% (4/4) |
|  |  | Wound |  | MDR infection: 100% |  |  | 0% (0/1) |

***Abbreviations****: MDR, Multi drug resistant*

*^a^Persistence*

Microbiological Cure or Eradication (Mixed/Unspecified Bacteremia), Reported Infection-specific Individual Patient Data

| **Author, Year** | **Study Design** | **Source** | **Pathogen type** | **Antibiotic resistance** | **Outcome Definition** | **Time Point** | **% (n/N)**  **Reporting Cure or Eradication** |
| --- | --- | --- | --- | --- | --- | --- | --- |
| **Retrospective cohort studies** | | | | | | | |
| Caston et al, 2017([14](#_ENREF_14)) | Retrospective cohort (single patient) | Otitis and mastoiditis and sepsis/septic shock | *Pseudomonas:* 100% (not further specified) | MDR infection: 100% | -- | 30 days (after therapy with C/T) | 100% (1/1) |
|  |  | Biliary (not further specified) and sepsis/septic shock |  |  |  |  | 100% (1/1) |
|  |  | Abdominal (not further specified) and sepsis/septic shock |  |  |  |  | 0% (0/1)^a^ |
|  |  | Abdominal (not further specified) and sepsis/septic shock |  |  |  |  | 100% (1/1) |
|  |  | Respiratory (not further specified) and sepsis/septic shock |  |  |  |  | 0% (0/1)^a^ |
|  |  | Respiratory (not further specified) and sepsis/septic shock |  |  |  |  | 100% (1/1) |
| Xipell et al, 2018([22](#_ENREF_22)) | Retrospective cohort (single patient) | Submandibular fasciitis | *Pseudomonas:* 100% (not further specified) | XDR infection: 100% | Defined as negative cultures for P. aeruginosa after 72 h of therapy when repeated cultures from the same source were available | -- | 0% (0/1) |
|  |  | UTI and deep surgical-site infection | *Pseudomonas*: 100% (not further specified), Enterobacter: 100%, K. pneumonia: 100%^f^ | PDR infection: 100% |  |  | 100% (1/1) |
|  |  | Septic shock due to cholangitis | *Pseudomonas*: 100% (not further specified), E. coli: 100%^b^ | XDR infection: 100% |  |  | 100% (1/1) |

***Abbreviations****: MDR, Multi drug resistant; PDR, Pan drug resistant; XDR, Extensively drug resistant*

*a Persistence;*

*b Poly microbial infection*

Mortality (Secondary Bacteremia), Reported Infection-specific Individual Patient Data

| **Author, Year** | **Study Design** | **Source** | **Pathogen type** | **Antibiotic resistance** | **Outcome Definition** | **Time Point** | **% (n/N)**  **Reporting Mortality** |
| --- | --- | --- | --- | --- | --- | --- | --- |
| **Retrospective cohort studies** | | | | | | | |
| Haidar et al, 2017([18](#_ENREF_18)) | Retrospective cohort (single patient) | Pneumonia | *P. aeruginosa:* 100% | MDR infection: 100% | Defined as P. aeruginosa if the patient died with signs and symptoms of infection, microbiologic or histological evidence of an active P. aeruginosa infection, and  if other potential causes of death were reasonably excluded | 90 days | 0% (0/1)^a^ |
|  |  |  |  |  |  | 30 days | 0% (0/1)^b^ |
| King et al, 2018([11](#_ENREF_11)) | Retrospective cohort | Pneumonia | *Pseudomonas:* 100% (not further specified) | MDR infection: 100% | -- | -- | 63% (5/8)^c^ |
|  |  | Urinary tract |  |  |  |  | 29% (2/7)^c^ |
|  |  | Intra-abdominal |  |  |  |  | 0% (0/4)^c^ |
|  |  | Wound |  |  |  |  | 100% (1/1)^c^ |

***Abbreviations:*** *MDR, Multi drug resistant*

*a Antibiotic resistance presented here not specific for bacteremia;*

*b Died within 90 days (not attributable);*

*c Died within 30 days (not attributable);*

Mortality (Mixed/Unspecified Bacteremia), Reported Infection-specific Individual Patient Data

| **Author, Year** | **Study Design** | **Source** | **Pathogen type** | **Antibiotic resistance** | **Outcome Definition** | **Time Point** | **% (n/N)**  **Reporting Mortality** |
| --- | --- | --- | --- | --- | --- | --- | --- |
| **Retrospective cohort studies** | | | | | | | |
| Xipell et al, et 2018([22](#_ENREF_22)) | Retrospective cohort (single patient) | Submandibular fasciitis | *Pseudomonas*: 100% (not further specified) | XDR infection | -- | -- | 100% (1/1) |

***Abbreviations****: XDR, Extensively drug resistant*
